# MEMS Switch Realities: Addressing Challenges and Pioneering Solutions

**DOI:** 10.3390/mi15050556

**Published:** 2024-04-23

**Authors:** Kurmendra, Saurabh Agarwal

**Affiliations:** 1 Department of Electronics and Communication Engineering, Rajiv Gandhi University, Rono Hills, Doimukh 791112, India; 2 Department of Electronics and Communication Engineering, Graphic Era Hill University, Dehradun 248002, India; saurabhagarwal@gehu.ac.in

**Keywords:** MEMS, switch, actuation, reliability, switching time, microstructure

## Abstract

Micro-Electro-Mechanical System (MEMS) switches have emerged as pivotal components in the realm of miniature electronic devices, promising unprecedented advancements in size, power consumption, and versatility. This literature review paper meticulously examines the key issues and challenges encountered in the development and application of MEMS switches. The comprehensive survey encompasses critical aspects such as material selection, fabrication intricacies, performance metrics including switching time and reliability, and the impact of these switches on diverse technological domains. The review critically analyzes the influence of design parameters, actuation mechanisms, and material properties on the performance of MEMS switches. Additionally, it explores recent advancements, breakthroughs, and innovative solutions proposed by researchers to address these challenges. The synthesis of the existing literature not only elucidates the current state of MEMS switch technology but also paves the way for future research avenues. The findings presented herein serve as a valuable resource for researchers, engineers, and technologists engaged in advancing MEMS switch technology, offering insights into the current landscape and guiding future endeavors in this rapidly evolving field.

## 1. Introduction

Micro-Electro-Mechanical System (MEMS) capacitive switches represent a cutting-edge advancement in electronic technology, revolutionizing the way we interact with devices. Unlike traditional mechanical switches, MEMS capacitive switches rely on the principles of capacitance to detect touch and trigger electrical responses. These switches are constructed with microscopic structures that can flex or deform when subjected to even the slightest touch. The capacitance changes resulting from this interaction are then translated into electrical signals, enabling seamless and reliable touch-sensitive controls. MEMS capacitive switches offer several advantages, including enhanced durability due to the absence of moving parts, reduced power consumption, and improved resistance to environmental factors such as dust and moisture. These switches find applications in various industries, from consumer electronics like smartphones and tablets to automotive touch panels and industrial control systems. As technology continues to evolve, MEMS capacitive switches are poised to play a pivotal role in shaping the future of human–machine interaction, providing users with intuitive and responsive touch interfaces.

Recent advancements in smart and portable systems demand miniaturized systems offering numerous functionality such as high speed, high resolution, ultra efficiency in performance, and most importantly, all these are achieved at the lowest possible cost [1,2,3,4,5]. MEMSs have brought revolutionary changes in the design of efficient systems, whether it is in the field of communication, sensing [6,7], biomedicals [8,9,10], safety instruments [11], food units [12], car manufacturing units [13,14], or sensor technologies. MEMS switches are utilized in these units along with actuators and sensors to offer great performances. Therefore, the design of MEMS switches for the aforementioned applications seeks high attention in the current era of technology. The performances achieved by these switches have been enumerated in the literature by researchers around the globe. The switches exhibit a compact form factor, enabling miniaturization, efficient integration of components, and low pull-in voltages. They demonstrate a high sensitivity to various actuation methods, contributing to their versatility and adaptability in different applications. Furthermore, the design often incurs low costs, making it economically viable. However, it is important to note that these switches may encounter reliability issues such as stiction, material wear, and fatigue, indicating a potential area for improvement in certain scenarios [15,16,17].

MEMS-based capacitive and ohmic contact switches represent innovative advancements in interface design. Integrating Micro-Electro-Mechanical Systems’ (MEMSs’) technology with capacitive sensing principles, MEMS-based capacitive switches offer compact, highly sensitive touch interfaces suitable for portable electronic devices and automotive applications [18]. Leveraging the precision of MEMS, these switches excel in detecting subtle changes in capacitance, ensuring reliable touch and proximity sensing. In contrast, MEMS-based ohmic contact switches combine MEMS technology with a direct physical contact approach, delivering a reliable and compact switch with tactile feedback [18,19]. These switches find applications in medical devices, consumer electronics, and industrial controls where a blend of precision and tactile response is essential [20]. Comparatively, MEMS-based capacitive switches provide a contactless interface with advantages like sleek design and resistance to wear, making them suitable for applications emphasizing hygiene and aesthetics. MEMS-based ohmic switches offer a tactile experience through direct physical contact, catering to environments where a clear indication of activation is crucial. The choice between these MEMS-based switch types depends on specific application requirements, considering factors such as sensitivity, form factor, and desired user experience [21,22].

MEMS-based acceleration, velocity, and pressure switches, employing either ohmic or capacitive contact types, epitomize advanced sensing technologies. In these switches, Micro-Electro-Mechanical Systems’ (MEMSs’) technology integrates with the principles of ohmic or capacitive sensing to deliver cutting-edge solutions for diverse applications. The ohmic contact types, relying on physical contact, offer tactile feedback and robustness, making them suitable for scenarios where a tangible indication of activation is essential, such as in automotive safety systems or medical devices [23,24,25]. Conversely, the capacitive contact types provide a contactless and precise sensing method, ideal for applications prioritizing hygiene, aesthetics, and resistance to mechanical wear, like in consumer electronics or environmental monitoring systems [26]. This versatile array of MEMS-based switches showcases the synergy of technology, addressing a spectrum of requirements across various industries with efficiency and innovation.

The researchers in [27] discuss MEMS switches and found that the performance achieved by the designed switches is better than their counterpart solid state-based switches. The solid-state switches suffer with issues such as greater area on chip, complex structure, high-voltage requirement and limitations for low-power very large-scale integration (VLSI) usages. The micromachined switches, however, offer multiple benefits by overcoming issues associated with solid state-based switches. In [28], Rebeiz et al. came out with a micromechanical switch design integrated with a transmission line which uses mechanical movement to operate as a switch. This was a successful switch design which can work for 0.1–100 GHz frequency ranges. They indicated that the MEMS switches can work with different actuation mechanisms such as electrostatic, magnetic, piezoelectric, and electro-thermal. However, designed switches were proposed to work with an electrostatic mechanism with an actuation voltage of 30 volts. The reliability of the switch was an important finding of the research study and it was found that the switch can be working reliably with 100 million to 10 billion cycles with desired performance.

SMA-based MEMS switches, leveraging the unique properties of Shape Memory Alloys (SMAs), represent an innovative approach to switch technology [16]. These switches utilize the reversible shape-changing characteristics of SMAs in response to temperature variations [29]. By applying heat, the SMA undergoes a phase transformation, enabling the switch to toggle between on and off states as it establishes or breaks electrical contact [30]. This design offers a low-power, reliable, and compact switching mechanism, making it advantageous for applications with limited space. SMA-based MEMS switches find utility in diverse fields such as telecommunications, aerospace, and medical devices [31]. While their adaptability and reduced energy consumption make them promising, ongoing research focuses on addressing challenges related to response time to temperature changes and precise thermal control for further optimization of SMA-based MEMS switch technology [32].

Commercial MEMS switches have become pivotal components in various electronic devices, revolutionizing the landscape of user interfaces [33,34]. These switches leverage Micro-Electro-Mechanical Systems’ (MEMSs’) technology, with two prominent contact types, capacitive and ohmic. Capacitive MEMS switches, exemplified by touchscreens in smartphones and tablets, offer a contactless interface, providing a sleek and responsive user experience [35]. Their success is evident in the widespread adoption across consumer electronics, where the demand for intuitive touch interfaces is paramount. On the other hand, ohmic MEMS switches, as seen in automotive applications like airbag deployment systems, provide tactile feedback through direct physical contact [36,37]. Their success lies in critical safety functions, showcasing the reliability and durability of this technology in demanding environments.

The comparison of these technologies reveals a trade-off between contactless precision and tactile responsiveness. Capacitive switches excel in applications prioritizing hygiene, aesthetics, and responsiveness, making them ideal for personal devices. In contrast, ohmic switches shine in scenarios where physical feedback is crucial, ensuring a clear indication of activation, as seen in safety-critical automotive systems. The success of commercial MEMS switches lies in their ability to cater to a spectrum of needs across diverse industries, demonstrating the versatility and effectiveness of MEMS technology in modern electronic applications.

The detailed parameters and related issues are further discussed in the next sections.

## 2. Characteristics of MEMS Switches

MEMS switch performances are dependent on various parameters and proper considerations of these parameters improves the performance of the designed switches for specific applications. They are described.

### 2.1. Actuation Voltage

**Figure 1:**
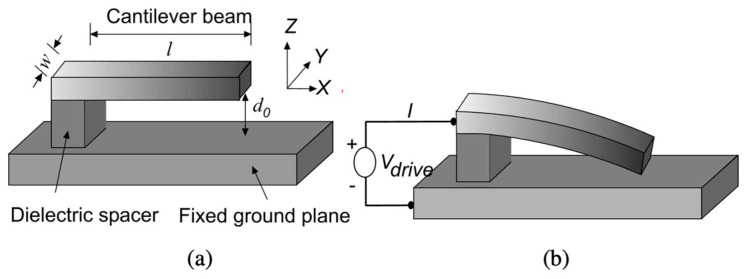
The effect of pull-in voltage in MEMS switch (**a**) when no voltage applied (**b**) when voltage $V_{drive}$ is applied [21].

The most important parameter in the designing of the MEMS switch is deciding the requirement of actuation voltage for a particular application. It is considered as the minimum voltage required to actuate the top beam membrane of the switch to being in the pull-in position. The actuation voltage is also termed as pull-in voltage. The pull-in voltage is defined as an electromechanical instability that is determined by the imbalance in the electrostatic forces and the mechanical restoring forces. This pull-in voltage affects various other parameters in the switch such as capacitance, switching speed, and reliability. Figure 1 shows the effect of pull-in voltage (*V_drive_*) in displacement of beam membrane of a cantilever-type MEMS switch.

The pull-in voltage in MEMS (Micro-Electro-Mechanical System) switches refers to the voltage at which the electrostatic force becomes strong enough to overcome mechanical restoring forces, causing the movable part (actuator) of the MEMS switch to collapse or move towards the stationary part. This collapse or movement leads to the switch transitioning from an “off” state to an “on” state. The pull-in voltage is a critical parameter in the operation of electrostatically actuated MEMS switches, and it is denoted as $V_{drive}$ in Figure 1. It can be mathematically expressed as [38]

(1) $$V_{drive}=\frac{8\;k\;{g_{0}}^{3}}{27\;\in_{0}\in_{r\;}(W\cdot w)}$$

where $V_{p}$, *k*, $g_{0}$, $\in_{0}$, $\in_{r}$, *W* and *w* are actuation voltage, spring constant, “air” height, permittivity of free space, relative permittivity of dielectric medium, CPW line width, and width of the membrane, respectively.

The magnetic, piezoelectric, electrothermal, and SMA mechanisms offer distinct advantages in addressing actuation voltage challenges in MEMS switches [39]. Magnetic mechanisms utilize efficient magnetic forces, allowing for low-actuation voltages and precise control [40]. Piezoelectric mechanisms leverage materials that generate electrical charges under mechanical stress, providing energy-efficient actuation at lower voltages [41]. Electrothermal mechanisms, based on resistive heating-induced material expansion, offer another low-voltage option with precise control [42]. Shape Memory Alloy (SMA) mechanisms utilize the reversible shape-changing properties of SMAs in response to temperature variations, enabling efficient actuation at relatively low voltages [43]. Each mechanism caters to specific application needs, balancing factors such as energy efficiency, precision, and ease of implementation to optimize actuation performance in MEMS switches.

The release voltage in electrostatic MEMS systems is a crucial parameter determining the minimum voltage required for the controlled release of movable components from their actuated positions [44]. This parameter plays a pivotal role in various MEMS applications, influencing the reliability and precision of the device operation. Engineers meticulously optimize the release voltage by considering factors such as electrode geometry, distance between electrodes, and material properties [45,46]. Striking the right balance in the release voltage is essential to avoid issues like excessive power consumption, potential damage, or incomplete release, ensuring the optimal performance and longevity of electrostatic MEMS devices.

### 2.2. Transmission Performance

The transmission performance of the MEMS switch is defined by three parameters, namely, insertion loss, return loss, and isolation. It is important to note that these three paraments of transmission are dependent on the frequency of operation, the capacitance formed between two plates, the distance between the two plates, and the dielectric properties.

#### 2.2.1. Insertion Loss

Insertion loss in MEMS (Micro-Electro-Mechanical System) switches is a parameter that characterizes the degradation in signal strength when the switch is in the “on” state. It quantifies the amount of signal power lost as it passes through the switch, and it is a key consideration in applications where signal integrity is paramount, such as in RF (Radio Frequency) and microwave circuits. Insertion loss is typically expressed in decibels (dB) and is denoted as IL [47,48]. The insertion loss in MEMS switches primarily arises from the resistive, capacitive, and inductive components introduced by the switch during the transmission of signals.

In the “on” state, when the switch is closed, these components contribute to the attenuation of the signal passing through the switch, leading to a reduction in signal amplitude. Several factors influence the insertion loss in MEMS switches, including the design of the switch, the materials used, and the operating frequency [49]. The geometry and dimensions of the switch components, such as the size of the contact pads and the gap between them, play a crucial role. Additionally, the type of actuation mechanism employed, whether electrostatic, electromagnetic, or thermal, can impact the overall insertion loss [50]. To mitigate insertion loss, researchers and engineers often focus on optimizing the switch design, exploring novel materials with low resistivity and dielectric losses, and employing advanced actuation mechanisms that minimize signal degradation. Furthermore, the choice of materials for the switch’s moving and stationary parts can influence the insertion loss, with low-loss dielectric materials and conductive elements aiding in maintaining signal integrity [51]. Accurate measurement of insertion loss is typically performed using network analyzers in controlled laboratory conditions.

The *S*-parameters (scattering parameters) of the MEMS switch, specifically $S_{21}$ representing the transmission coefficient from the input to the output port, are critical for evaluating insertion loss. It is mathematically described as [22]

(2) $$S_{21}=\frac{1}{1+\frac{j\omega C_{d}Z_{0}}{2}}$$

where symbols are denoted as downstate capacitance (*C_d_*), resonance frequency of the switch (*ω*), and characteristics impedance (*Z*_0_) of CPW transmission line.

In summary, insertion loss is a fundamental metric in assessing the performance of MEMS switches. Engineers and researchers aim to minimize insertion loss to ensure optimal signal transmission through these switches, particularly in applications where signal quality and integrity are paramount. Optimization efforts often involve a holistic approach, considering the design, materials, and actuation mechanisms to strike a balance between switch performance and signal preservation.

#### 2.2.2. Return Loss

Return loss in MEMS (Micro-Electro-Mechanical System) switches is a performance parameter that describes the amount of power reflected back to the source due to impedance mismatch. It is commonly expressed in decibels (dB) and is an essential consideration in the design and evaluation of MEMS switches, especially in RF (Radio Frequency) and microwave applications. Return loss (RL) is mathematically defined as

(3) $$S_{11}=\frac{-j\omega C_{u}Z_{0}}{2+j\omega C_{u}Z_{0}}$$

where the parameter denotes an upstate capacitance as *C_u_*. A higher return loss value indicates a lower amount of reflected power, signifying a better match between the source and the load [52]. Conversely, a lower return loss indicates a higher reflected power and a less-efficient impedance match.

The factors influencing return loss in MEMS switches include switch design, material properties, actuation mechanism, frequency of operation, and surface roughness [53]. The design of the MEMS switch, including its geometry, size, and actuation mechanism, can influence the impedance matching and, consequently, the return loss. The choice of materials for the MEMS switch components affects their electrical properties, such as conductivity and dielectric constant, which, in turn, impact return loss [54]. The method used to actuate the MEMS switch (e.g., electrostatic, thermal) can affect the switch’s electrical characteristics and, consequently, the return loss. Return loss is often frequency dependent. MEMS switches designed for specific frequency bands should be optimized for a low return loss within those bands [55]. The surface roughness of the MEMS switch’s conductive elements can contribute to reflections and impact the return loss, especially at higher frequencies.

Return loss is typically measured using network analyzers in a laboratory setting. The S-parameters (scattering parameters) are commonly used to characterize the performance of RF and microwave components, including MEMS switches. The return loss is represented by $S_{11}$, which is the reflection coefficient at Port 1 of the device. In summary, return loss is a critical parameter in the evaluation of MEMS switches, especially in RF and microwave applications. Design considerations and optimization are essential to achieving low return loss and, consequently, improved performance in terms of power efficiency and signal integrity.

#### 2.2.3. Isolation

Isolation in an MEMS (Micro-Electro-Mechanical System) switch refers to the capability of the switch to prevent unwanted coupling or crosstalk between different ports or paths within the device. It is a crucial parameter, particularly in applications involving RF (Radio Frequency) and microwave signals, where maintaining signal integrity and preventing interference are paramount [56]. The isolation of an MEMS switch is denoted as ISO and is measured in decibels (dB). The isolation between two ports is essentially the reciprocal of $S_{21}$ and is given by

(4) $$Isolation\;(ISO)=-|S_{21}|$$

In practical terms, isolation quantifies the extent to which signals directed to one port of the switch are isolated from signals at other ports when the switch is in the “off” state. The goal is to minimize leakage or coupling between ports, ensuring that signals intended for a specific path do not interfere with or affect signals in other paths [57]. This is especially critical in communication systems and RF circuits, where unwanted signal interactions can lead to the degradation of system performance. The isolation in an MEMS switch is typically evaluated by measuring the power leaking from one port to another, expressed as a ratio to the incident power at the input port. The higher the isolation value, the better the switch can suppress unwanted signals, resulting in improved performance and reliability [58]. Achieving high isolation involves careful design considerations, including optimizing the geometry of the switch components, employing appropriate materials with low-crosstalk characteristics, and selecting suitable fabrication techniques. One common method to enhance isolation is to physically isolate the different paths within the switch using well-designed structures and configurations. This can include implementing shielding or employing specific geometries that minimize electromagnetic coupling between adjacent paths. Additionally, advanced actuation mechanisms, such as electrostatic, electromagnetic, or thermal actuation, can be employed to further isolate paths and reduce interference [59]. Engineers and researchers continually strive to improve isolation in MEMS switches to meet the increasingly stringent requirements of modern communication systems. This involves a multidisciplinary approach that combines expertise in MEMS design, materials science, and RF engineering [60,61]. As MEMS technology advances, achieving higher levels of isolation becomes a key factor in ensuring the reliability and performance of MEMS switches in diverse applications.

### 2.3. Capacitance Ratio

The capacitance ratio in MEMS (Micro-Electro-Mechanical System) switches is a parameter that describes the relationship between the capacitance in the “on” state to the capacitance in the “off” state. It plays a significant role in determining the performance of MEMS switches, particularly in RF (Radio Frequency) and microwave applications. The capacitance ratio is denoted as ratio Cratio and is defined as

(5) $$C_{ratio}=\frac{C_{on}}{C_{off}}$$

*C_on_* is the capacitance in the “on” state (when the MEMS switch is closed) and *C_off_* is the capacitance in the “off” state (when the MEMS switch is open).

A high capacitance ratio is desirable as it indicates that the capacitance in the “on” state is significantly higher than in the “off” state [62]. This is crucial for minimizing signal loss and insertion loss when the MEMS switch is in the closed position.

The capacitance ratio affects the switching speed of the MEMS switch. A lower capacitance in the “on” state allows for faster charging and discharging of the capacitive elements, enabling quicker switching times; however, it is desired to have a high capacitance ratio for a better isolation profile. In applications where power consumption is a critical consideration, a lower capacitance ratio is advantageous. Lower capacitance in the “on” state results in reduced power consumption during switching [63]. Achieving a high-capacitance ratio is essential for maintaining good impedance matching, especially in RF and microwave circuits. It ensures that the MEMS switch presents a low impedance in the “on” state and a high impedance in the “off” state. The capacitance ratio is typically measured experimentally using impedance analyzers or network analyzers. By applying a voltage or a test signal and measuring the resulting capacitance in both the “on” and “off” states, the capacitance ratio can be determined [64,65,66,67]. In summary, the capacitance ratio is a key parameter in the design and evaluation of MEMS switches. Achieving a low capacitance in the “on” state and a high capacitance in the “off” state is essential for optimizing the switch’s performance in terms of signal integrity, speed, and power consumption.

### 2.4. Contact Resistance

Contact resistance is a critical consideration in MEMS ohmic contact switches, influencing the performance and efficiency of these devices [68]. The Figure of Merit (FOM), expressed as FOM = *R_on_*/*C_off_*, serves as a metric to evaluate the quality of the switch, where Ron represents the contact resistance, and Coff represents the off capacitance [69]. Achieving a low FOM is essential, as it indicates both low contact resistance and low off capacitance, leading to enhanced switch performance [70]. In the context of ohmic contact switches, achieving a low-contact resistance poses significant challenges. One major challenge lies in the selection of appropriate contact materials. The materials must exhibit low resistivity and maintain stability under the operating conditions of the MEMS switch [71].

Additionally, optimizing the contact force is crucial. The contact force influences the reliability and operational voltage of electrostatic devices, as excessive force may lead to wear or damage, affecting the contact resistance [72]. Reliability is a paramount concern in ohmic contact switches, as these switches may experience wear and tear over time due to mechanical cycling [73]. Furthermore, the continuous improvement of contact materials and manufacturing processes is necessary to overcome challenges associated with contact resistance. Researchers and engineers are actively working to develop innovative solutions and materials to achieve low-contact resistance and improve the overall performance of MEMS ohmic contact switches [74,75]. Addressing these challenges is essential for advancing the reliability and efficiency of these switches in various applications.

### 2.5. Switching Time

The switching performance of the micromechanical switches is another important parameter. The switching time can be defined as the time taken by the switch to create open (off) and short circuit (on), in other words, the time taken by the switch to transmit the portion of the signal and to isolate the signal from input. The switching analysis also relates to the reliability of the switch. The switching performance is limited by various parameters such as the stiction problem, dielectric charging and discharging, aging effect, temperature, and material used for making the top and bottom electrodes of the switch. The expressions and methods for finding the switching time for a designed MEMS switch can be complex, as discussed in articles [76,77].

### 2.6. Reliability

The reliability of the MEMS switches is governed by many parameters such as the fabrication process and use of different materials such as materials for electrode, dielectric, transmission lines, charging phenomenon in the dielectric etc. [78,79,80]. Thus, controlling these parameters would result in improved reliability of the switch. The improving life cycle of the switch also improve the reliability. It has been found that most of the fabricated switch has reliability issues and many techniques are employed from time to time to improve the reliability in which some techniques such as use of metal alloys, use of stack structures, use of proper dielectric materials, and controlled fabrication processes are most common and effective [81,82,83].

The hot switching, or the act of making and breaking electrical connections in an MEMS switch while the current is flowing, can significantly impact the reliability of both capacitive and ohmic switches [84,85]. In capacitive switches, hot switching can lead to issues such as dielectric breakdown and electrode degradation due to the high electric fields generated during the switching process [86]. This can result in increased contact resistance and reduced switch reliability over time.

For ohmic switches, hot switching poses challenges related to the mechanical wear and thermal stress experienced by the contacts during the switching events [87]. High currents flowing through the contacts can cause localized heating, leading to material fatigue and wear [88]. This wear and tear may result in increased contact resistance, reduced switch lifetime, and potential device failure.

Research perspectives on addressing the impact of hot switching in MEMS switches involve investigating advanced materials with improved thermal and mechanical properties [89]. Exploration of novel materials, such as nanocomposites or materials with self-healing properties, aims to enhance the durability of switches under hot switching conditions [90]. Additionally, optimizing the switch design to minimize current densities, incorporating protective coatings, and developing innovative contact materials are areas of active research to improve the reliability of both capacitive and ohmic MEMS switches during hot switching events [91]. By advancing materials and design strategies, researchers aim to extend the lifespan and robustness of MEMS switches, ensuring their reliable performance in demanding applications [92,93].

The typical failure modes in MEMS switches can be diverse, involving mechanical, electrical, and material-related issues. Below are some common failure modes and potential solutions as illustrated in Table 1.

Table 2 provides a generalized overview, and specific failure modes and solutions can vary based on the MEMS switch design, application, and operating conditions. The MEMS switch reliability is a multidisciplinary challenge, and ongoing research aims to explore new materials, design strategies, and fabrication techniques to address these failure modes and enhance the overall robustness of MEMS switches across various applications.

### 2.7. Power Handling/Self Actuation Voltage

In RF systems utilizing MEMS switches, induced voltage can significantly impact performance. Unintended actuation or release, signal distortions, and switching speed variations may occur due to induced voltages from RF signals or external electromagnetic fields [41,94]. This interference can lead to switching errors, signal integrity issues, and reliability challenges, affecting the overall functionality of the RF system [95]. To address these concerns, designers implement shielding methods, filtering techniques, and optimized switch geometries to mitigate the effects of induced voltages [73,96,97]. The ongoing focus on research and advancements in MEMS technology aims to develop solutions that enhance the robustness and reliability of MEMS-based RF switches, particularly in the presence of induced voltages from the RF environment [98].

The current and electrothermal response constitute the second facet of power handling in MEMS switches, presenting distinct challenges for capacitive and ohmic switch types [99]. In capacitive switches, the challenge lies in managing the high current densities during actuation, which can lead to Joule heating effects and potential device failure [100]. To address this, advanced materials like liquid metals or semi-metals, including Carbon Nanotubes (CNTs), are explored. These materials exhibit superior thermal conductivity, mitigating the impact of Joule heating and enhancing the overall electrothermal response in capacitive switches [101,102,103]. Conversely, ohmic switches face challenges associated with current-carrying capacity and heat dissipation. High currents passing through ohmic contacts can result in elevated temperatures, affecting the switch’s reliability and operational lifespan [104]. Liquid metals and CNTs are also investigated in ohmic switches to enhance their current-carrying capabilities and improve heat dissipation, thereby addressing challenges related to power handling [105]. The unique thermal and electrical properties of these materials contribute to more efficient power distribution and improved electrothermal performance in both capacitive and ohmic MEMS switches. Ongoing research aims to optimize the integration of liquid metals and CNTs to overcome the current-related challenges, ensuring robust power-handling capabilities in MEMS switch technologies [106,107].

## 3. Types of MEMS Switches

The MEMS switches are classified on the basis of mechanical structure, actuation method, circuit configuration, and contact type [108]. The different mechanical structures such as cantilever, clamped–clamped, and diaphragm structures are the most utilized structures [109,110]. The meanders of different shapes and holes with different shapes have been introduced to further improve performance of the designed switches [111,112,113,114]. In MEMS switches, the most utilized actuation methods are electrostatic, electrothermal, electromagnetic, piezoelectric, and SMA [97,115,116,117,118,119]. These switches are used in series, shunt, or mixed configurations with transmission lines of test systems as well as communication systems. The MEMS switches are also classified on the basis of contacts made between the top metal beam and bottom electrode. If the contacts are made with two metal plates for the purpose of switching, it is known as the ohmic contact switch and if indirect contacts (used of dielectrics) are made between two metal electrodes, it is known as the capacitive MEMS switch. The detailed discussion on different types of switches is presented in further sections.

### 3.1. Geometric Constraints

**Figure 2:**
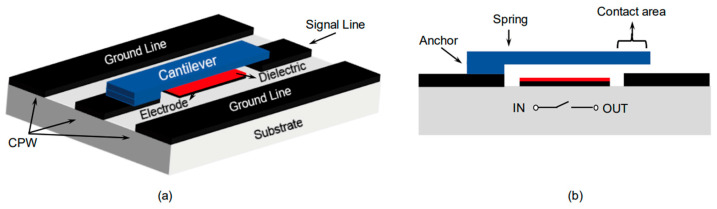
(**a**) Cantilever beam with coplanar waveguide line (**b**) side cross-sectional view of the cantilever switch [120].

Cantilevers are mechanical structures and are widely used in fabricating mechanical devices; they are very useful in offering good performances as mechanical structures. When they are used in MEMS switches, they offer various advantages such as good sensing properties, low pull-in voltage, good switching speed, and good RF performances. However, their use is limited in specific frequency applications because of reliability issues and less life span. The structure of a cantilever-based MEMS switch is presented in Figure 2.

**Figure 3:**
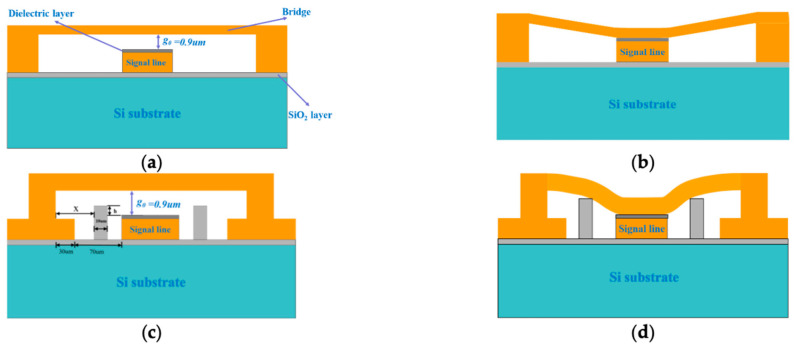
(**a**) Clamped–clamped structure RF MEMS switch OFF, (**b**) RF MEMS switch ON, (**c**) the up state of the RF MEMS switch, (**d**) the down state of the RF MEMS switch [121].

Another mostly utilized structure in mechanical devices is the clamped–clamped structure. Few MEMS switches use clamped–clamped structures as movable mechanical elements of the switch and this movable structure is clamped at both the ends. Thus, the MEMS switches are also named clamped–clamped MEMS switches. The clamped–clamped MEMS switch structure has various advantages such as high frequency (>10 GHz) of operation, good switching characteristics, and a reliable and better life span. However, the life span of these switches also depends on other parameters of the switch such as contact type and material used for electrodes. The clamped–clamped switch is presented in Figure 3.

**Figure 4:**
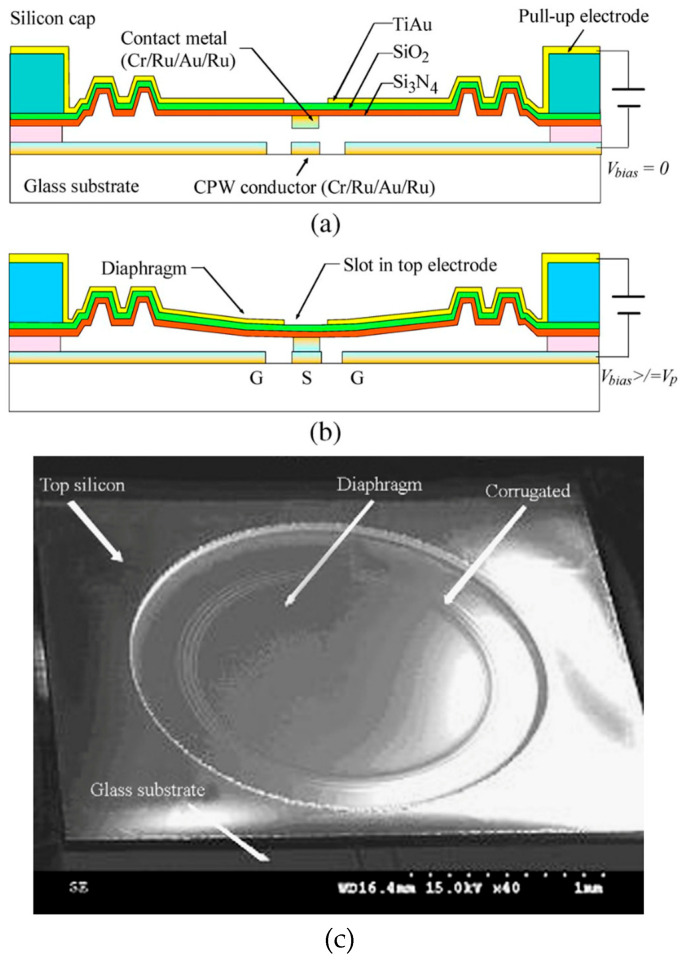
(**a**) Diaphragm structure MEMS switch with coplanar waveguide line (*V_bias_* = 0); (**b**) diaphragm structure MEMS switch (*V_bias_* > *V_p_*) [44]; (**c**) SEM image of circular diaphragm structure MEMS switch [122].

Most MEMS switches are found to be designed in either the cantilever or clamped–clamped structure. The dimensions of the top electrodes in both the cases are the same but their placement orientation is different; as for cantilevers, one end is kept free and for the clamped–clamped structure, both ends are fixed. The less popular structure for moving electrodes in switches are diaphragms. A diaphragm serves as a structural component responsible for transmitting lateral loads to the vertical resisting elements within a structure. In general, the length and width are in millimeters and thickness is in microns. A diaphragm structure-based MEMS switch is shown in Figure 4.

Figure 4a shows a multilayered diaphragm structured MEMS switch without application of electrostatic voltage and Figure 4b shows the effect of electrostatic potential *V_bias_* equal to and more than the pull-in voltage *V_p_*. Figure 4c shows the SEM image of a fabricated circular diaphragm MEMS switch structure.

### 3.2. Actuation Mechanisms

The MEMS switches are also classified on the basis of actuation methods used. The actuation mechanisms which are generally used for MEMS devices are electrostatic, electromagnetic, electrothermal, and piezoelectric [123,124]. The structure of these switches can be a cantilever, clamped–clamped, or diaphragm structure, but the actuation methods would be different. There are several advantages of choosing an appropriate actuation mechanism among the available actuation mechanism. The advantages and disadvantages of these actuation techniques are listed in Table 1.

### 3.3. Resistive Series Switches

**Figure 5:**
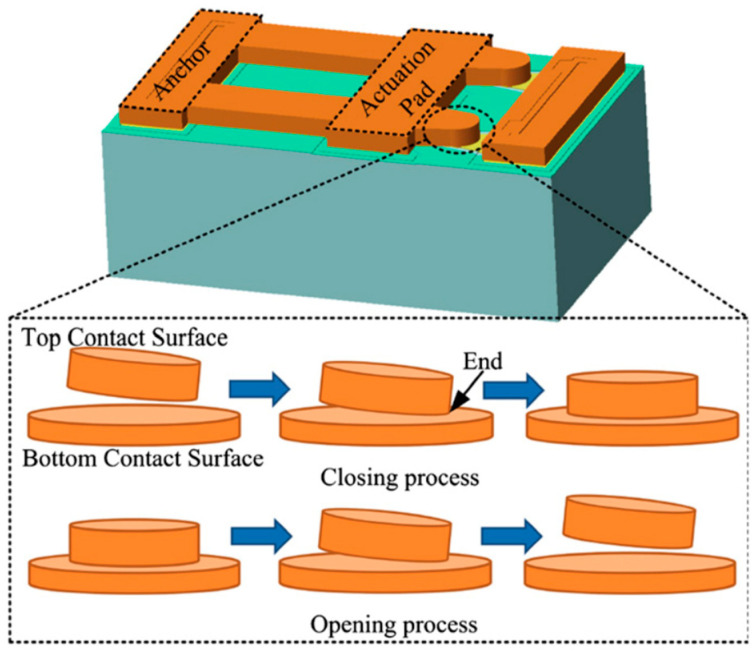
Resistive (metal-to-metal contact) MEMS witch with CPW line and fabricated top view of MEMS capacitive switch [125].

MEMS switches make contact for open and short circuits with coplanar waveguide lines upon actuation. These contacts can either be made metal-to-metal or metal–dielectric–metal configurations. The switches, which make metal-to-metal contact upon actuation, are known as resistive switches. A design of resistive MEMS capacitive switches is given in Figure 5. The top-beam electrode with two ends of the cantilever structure is shown, which is making contact with the bottom electrode signal line (yellow color). The enhanced ends of one of the cantilever ends is shown in the figure, demonstrating the closing (on) upon actuation and opening (off) process upon removal of actuation method of the switch. The metal-to-metal contacts on each use reduces the life span of the MEMS switches due to metal removal and subsequent change in resistance is seen. The metal-to-metal contact or resistive switches offer certain advantages of offering a low pull-in voltage requirement, and good RF performance. They, however, suffer from reliability issues, as after multiple actuations, the metal ends do not offer the required performance in opening and closing functions.

### 3.4. Capacitive Shunt Switches

**Figure 6:**
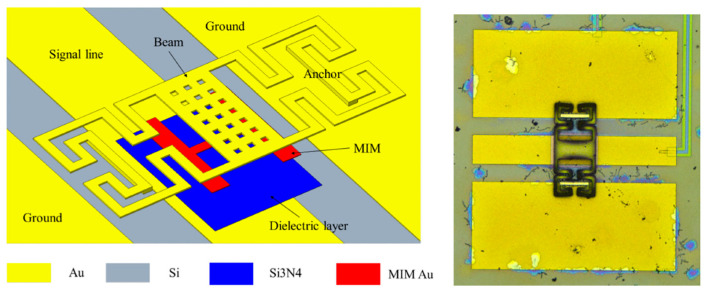
MEMS capacitive switch with CPW line and fabricated top view of MEMS capacitive switch [126].

The capacitive switches can be used in MEMS devices in shunt or series configurations. The nomenclature of capacitive switch comes from the capacitances formed in the switch structure and dependency of the RF performance of these switches on capacitance. The structure of capacitive MEMS switch is depicted in Figure 6. The capacitive switches have layers of a moving electrode, a dielectric, and a fixed electrode. The electrodes can be metal or semiconductor, thus the switch forms a capacitor. The switches can be either in cantilevered from or clamped–clamped forms. The capacitive switches have advantages over resistive switches as resistive switches suffer from reliability issues and have low life cycles but capacitive switches do not make direct contact with the bottom metal electrode (CPW signal line) which makes a capacitive short circuit during operation which improves the reliability and life cycle as compared to resistive switches.

A detailed discussion on the issues related to MEMS switches and methods to overcome such issues are presented in Section 4.

## 4. Key Issues Related to MEMS Switches and Improvement Techniques

The aforementioned switches, despite offering various advantages, have certain limitations when they are designed for the desired frequency of operation and desired applications. It is therefore important to discuss issues related to MEMS switches and methodology to overcome such issues. The limitations of some MEMS switches are a high actuation voltage requirement, low switching speed, stiction problems, charging of dielectrics, complex structures, reliability issues, and fabrication processes [127,128,129].

### 4.1. High Actuation Voltage

**Figure 7:**
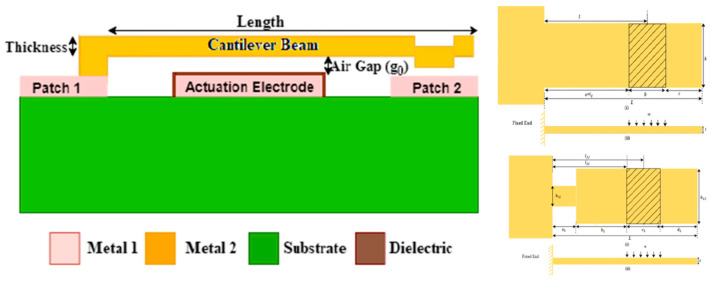
The design of the ohmic DC RF MEMS switch and geometric profile of the designed cantilever switch to overcome mechanical failure due to reduced stiffness [130].

The demand of reducing power requirement for low power very large-scale integration (VLSI) devices is an important factor. The MEMS switches are used with VLSI components and this reduced pull-in or actuation voltage becomes very important and challenging for MEMS component designers. The literature on MEMS switches suggests a mechanism to overcome the issue and fabricate switches offering a low-power requirement. Kasambe et al. [130] designed an ohmic contact RF MEMS switch to offer low pull-in voltage for 20 kHz applications. The design of the switch considers the cantilever and the pull-in voltage is reduced by lowering the cantilever stiffness constant. The challenges involved in the design is that upon lowering the stiffness of the materials, the switch mechanical performance such as stress carrying capacity, stiffness, and resonance frequencies decrease. The authors addressed the issues related to mechanical failure of the switch by optimizing the model such that the designed switch can easily withstand a 11.20 V low pull-in voltage without failure. The design of the switch is presented in Figure 7.

**Figure 8:**
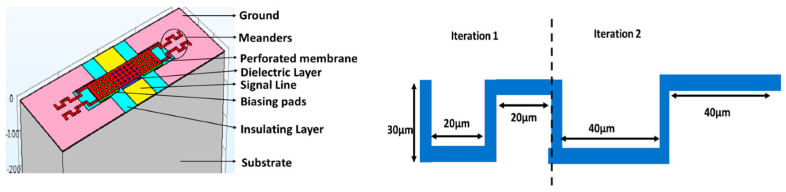
The design of the capacitive RF MEMS switch and meander structure to offer low pull-in voltage [131].

In A. Kumar et al. [131], the researchers used two prospective studies to reduce the pull-in voltage requirement for a designed MEMS switch. The first prospective study was to use a square shape on the 60% area of the suspended electrode and the second was to use a meander structure at the end in place of a uniform shape, as shown in Figure 8. The designed switch offers reduced pull-in voltage because of a reduction in spring constant of the top suspended beam electrode due to perforations and meanders used in the structure. The pull-in voltage was reduced to as low as 1.8 V for the structure presented. The designed switch could offer 30–300 GHz range 5G applications. The challenge herein involved in the design of the switch is that more fabrication processing steps and a huge cost along with controlled mechanical performance need to be achieved.

**Figure 9:**
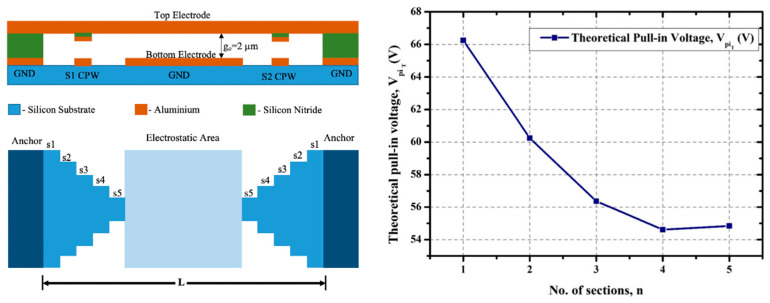
The design of the capacitive RF MEMS switch and meander structure to offer low pull-in voltage [133].

In Deng et al. [132], the authors propose a new type of spring support structure and match the width of the suspended electrode with a signal pad, as shown in Figure 6. This method also reduces the pull-in voltage on the principle of reducing the spring constant of the beam by using a perforated structure with H-shaped hinges. The output of the research validates that the switch can offer a pull-in voltage as low as 4.4 V for a beam thickness of 1 μm. The researchers in [133] presented an MEMS capacitive switch with silicon nitride dielectric involving a varying section fixed–fixed beam as show in Figure 9. The varying section of the clamped–clamped beam reduces the spring constant of the beam and offers low pull-in voltage. The graph in Figure 9 shows that the increase in the number of sections used for forming the beam reduces the pull-in voltage by reducing the spring constant to a greater extent while maintaining the strength of the beam for the designed applications. The switch presented in Figure 9 offers a pull-in voltage less than 56 V for five varying sections in the switch.

**Figure 10:**
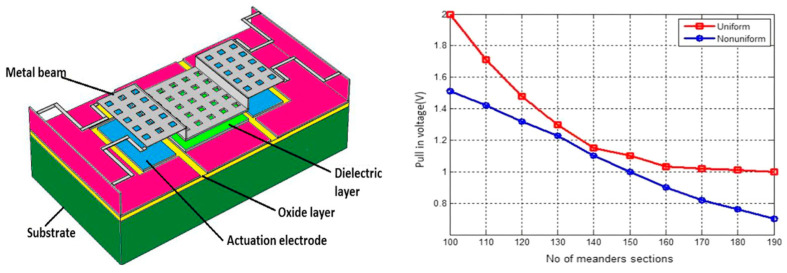
The design of the capacitive RF MEMS switch and serpentine meander structure to offer low pull-in voltage [134].

In Sravani et al. [134], a serpentine meander structure is used at the fixed ends of the top electrode of an MEMS capacitive switch as shown in Figure 10. It is notable that the authors used uniform and non-uniform meanders in their study for reducing the pull-in voltage. The structure also used square-shaped perforations on the main area of the electrode which touches the dielectric to make the capacitive short during off conditions of the switch. The introduction of square perforations and serpentine meander structures offers a pull-in voltage value of 1.1 V and from the curve, it is noticeable that for uniform and non-uniform meanders, the pull-in voltage reduces as the number of meanders are increased. The effects of number of meander sections are more prominent in non-uniform structures as compared to the uniform sections.

**Figure 11:**
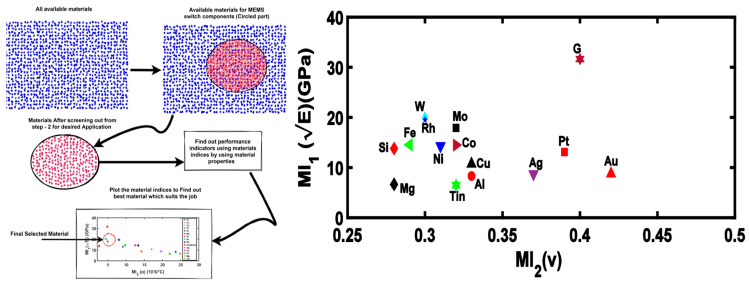
Ashby’s methodology to find out materials for different sections of the MEMS switch and plot of two indices, Young’s modulus and Poisson’s ratio [135].

The actuation voltage or pull-in voltage requirement can be lowered to a great extent by means of employing a beam with low spring coefficient materials, using perforations on the beam, different shaped hinges, meanders of different shapes, and using non-uniform sections in the beam hinges as discussed above. However, sometimes the cost of fabricating non-uniform meanders and hinges limits the fabricating of such switches. The other important parameter to reduce the pull-in voltage is the selection of proper material for the beam electrode. The selection of such materials becomes an important task for designers of MEMS switches, and therefore, researchers across the world have provided different techniques for the selection of beam electrode materials. In [135], the authors recommended methods for a selection of clamped–clamped beam material using Ashby’s methodology as shown in Figure 11 which uses material indices to decide the best material for the selection of the material out of available materials. The plot between two material indices, Young’s modulus and Poisson’s ratio, has been depicted in Figure 10. These two material indices are related to stiffness of the materials and it has been found that the materials having low values of Young’s modulus and a high Poisson’s ratio offer less stiff material. Thus, from the curve, it has been found that Au, Ag, and Pt can be a good choice of material for offering low pull-in voltage.

### 4.2. Dielectric Charging and Stiction Problem

Stiction is considered a major problem in the operation of MEMS switches for different applications and limits the use of these MEMS switches [136,137]. As the electrodes are miniaturized metal plates, when they stick together, the effective force causes the surfaces of the electrode to remain stuck together and causes a malfunction of the device by not releasing the movable electrode. The capillary force (due to condensed water between the surfaces), the molecular van der Waals force, micro-welding, and electrostatic forces (charging) are common causes of stiction in MEMS [138]. The stiction is more dominant in the devices operating at high temperature ranges as the hot metal plates get stuck together due to expansion of the surface of the electrodes. The metal-to-metal contact switches are mostly suffered structures from stiction issues due to migration of metal particles upon the cyclic application of MEMS devices [139]. The capacitive MEMS switches use dielectric materials and it has been found that the high-k dielectric materials are subject to charging during application of actuation voltages. Due to charging of high-k dielectric materials, the metal electrode gets stuck to the dielectric layers and cannot release an electrostatic force to come back to the original positions [140]. The remedy to avoid stiction in metal-to-metal contact switches is to use moderate stiff material, and in capacitive MEMS switches, to use low-k or moderate dielectric constant materials such as Si_3_N_4_ and SiO_2_ [141].

In T. Singh et al. [142], the researchers proposed the use of nonmetallic membrane for avoiding stiction in MEMS-resistive switches. The metallic electrodes introduce a stiction problem in the long duration of use due to a degradation in the metal atoms from the surfaces. The proposed method has been found to be an effective solution to avoid stiction and improve reliability of the MEMS switches. Another solution to mitigate the stiction problem in MEMS switches is the use of harder metallic materials [143]. However, use of such materials causes issues of pull-in voltage requirements and switches the speed because of more of a stiffness constant for a harder metal.

In Md Ataul Mamun et al. [144], the researchers bring a solution to the stiction problem in metal contact switches. The authors developed an analytical model to estimate the stiction force in the electrostatically actuated Si nanoelectromechanical cantilever relays with a Pt contact metal MEMS switch. They demonstrated that the stiction force is dependent on contact resistance and drained to the source current (contact current). The stiction force, primarily to the metallic bonding force, is directly proportional to the contact current which can be tuned by tuning the contact resistance.

The stiction problems are one of the main causes of failure of MEMS switches which limits the use of MEMS switches. The aforementioned approaches can be useful in mitigating stiction issues in the MEMS switches.

Another important problem related to MEMS switches which needs attention of the RF engineers is dealing with dielectric charging phenomenon in the MEMS switches. It happens in the pull-down state where charges are injected from the metal electrode (clamped–clamped) into the dielectric layer under electric fields usually in the range of MV/cm at the contact points [145]. The dielectric charging mainly occurs in electrostatic MEMS switches due to high actuation voltages which causes a random distribution of electric charges on the bottom electrode due to polarity of the applied voltages [66]. Due to this phenomenon, we see abrupt change in pull-in voltage and functionality of the device changes, the releasing force is also affected during the release time upon removal of the applied voltage [146]. The dielectric charging is considered one cause of the stiction issue in electrostatically actuated MEMS switches [68]. In addition to the above phenomenon, the dielectric charging happens in MEMS switches due to other reasons such a charge distribution on the dielectric materials, and interface charges at the dielectric–metal interface [147].

**Figure 12:**
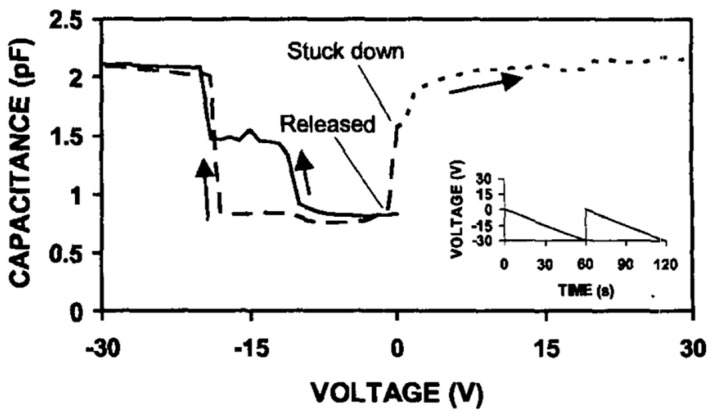
The comparison of the pre and post application of the actuation voltage and charging pattern [148].

The dielectric charging restricts the performance of an MEMS switch; therefore, the researchers have introduced methods to reduce dielectric charging in the MEMS Capacitive switches to offer improved performance. Xiaobin Yuan et al. [148] performed a study on an MEMS capacitive switch charging for polarized positive and negative pull-in voltages. The comparison of the positive and negative applied voltage on the charging of the capacitive switch is presented in Figure 12. It can be seen that for application of a voltage sweep of 0–30 V positive, the charges are created on the dielectric and the suspended beam electrode is stuck due to this charging and upon the application of a negative voltage, the electrode is released and discharging happens due to a decrease in charge during application of polarized voltages. 

In Muhammad Zubair et al.’s study [149], the dielectric charging phenomenon due to surface roughness in capacitive MEMS switches causes a reliability issue. It was discussed that the two types of charging that occur in the MEMS capacitive switch are as follows: (a) injection or contact charging; it occurs basically due to contact of the suspended beam electrode with the dielectric during a pull-in state and (b) the induced charging; mainly provoked by the translation of free intrinsic charges during the up state of the switch. The induced charging through field emission is the major cause of charging during the pull up of the switch. Thus, the charging is also because of the air gap between the two electrodes. An FEM-based study was performed and it was observed that the impact of surface roughness increases with a decrease in air gap between two electrodes, Therefore, it was recommended that the proper air gap needs to be maintained to control the aforementioned charging phenomenon in the capacitance MEMS switches.

R.W. Herfst et al. [150] measured a stressed device’s surface potential over time in order to better understand how a permanent charge is trapped in the dielectric (silicon nitride), and the results were obtained which reveal that the surface potential not only gradually declines but also initially rises at the minima, indicating that charge moves from the maximum to the minimum. The surface potential eventually decreases at the minima as well. This demonstrates that charge leaks back into the bottom electrode over a longer period of time and that diffusion initially dominates changes in the surface potential. Later, J. Theocharis et al. [151], for the first time shows an MEMS capacitor’s potential distribution, even when the field emission leakage current is present and the bottom electrode is covered in thin dielectric film. The research also showed how this process builds up dielectric charging. The investigation is based on analyzing the transport mechanisms in MIM capacitors and obtaining current–voltage characteristics in clockwise and counterclockwise loops. The same process is used to keep track of MEMS capacitors’ dielectric charging buildup during field emission. The voltage drops across the dielectric film, the gap, and their dependence on the flowing current are all determined using the data of perfect current–voltage characteristics in both MIM and MEMS.

The proper selection of material for the dielectric also controls the dielectric charging phenomenon in capacitive switches. In [135,152], the authors investigated a database of dielectric materials to recommend materials which offer low dielectric charging for highly resistive material on the facts that the highly resistive material reduces the chance of decaying the polarization, thus improving the dielectric charging problem.

### 4.3. Reducing Switching Time and Challenges

The switching time of MEMS (Micro-Electro-Mechanical System) switches is a crucial performance parameter that significantly influences their effectiveness in various applications. MEMS switches are known for their rapid response and low latency, making them suitable for applications where quick and precise switching is essential. The switching time of MEMS switches is typically in the microsecond to nanosecond range, depending on the specific design and technology used. The inherent mechanical nature of MEMS switches allows for swift actuation, enabling them to toggle between states with minimal delay. This quick switching time is particularly advantageous in telecommunications, RF (Radio Frequency) applications, and other scenarios where high-speed signal processing is paramount. As technology continues to advance, ongoing research and development efforts are aimed at further optimizing MEMS switch designs to achieve even faster switching times, opening up new possibilities for their integration into diverse electronic systems.

The switching time of MEMS (Micro-Electro-Mechanical System) switches is a critical aspect that has garnered considerable attention in research and development due to its impact on the performance and reliability of these devices as presented in Table 3. The squeeze film damping is a critical factor influencing the switching time of MEMS switches, where the resistance generated by the thin film of gas or liquid between moving and stationary parts can significantly impact the overall performance [153]. The switching time, representing the transition duration between different states of the MEMS switch, is influenced by the interplay of mechanical, electrical, and damping-related factors. Squeeze film damping contributes to increased effective mass and resistance, potentially slowing down the motion of the switch. Engineers employ design strategies to optimize gap distance, pressure, and other parameters to mitigate the impact of squeeze film damping on switching time, ensuring an efficient and reliable operation of MEMS devices [154,155].

While MEMS switches offer rapid and efficient switching, several challenges and issues are associated with their switching time. One primary concern is the potential for variability in the switching time across different MEMS devices, which can arise from manufacturing tolerances, material properties, and environmental factors. This variability can lead to inconsistencies in performance and hinder the widespread adoption of MEMS switches in certain applications. Moreover, the long-term reliability of MEMS switches, particularly in high-frequency and high-power environments, remains a topic of investigation. Issues such as stiction, wear and tear, and fatigue can affect the switching time over the operational lifespan of the device. Researchers are actively addressing these challenges through advancements in materials, fabrication techniques, and innovative designs to enhance the overall reliability and consistency of MEMS switches, ensuring their viability in diverse technological applications. As the field progresses, a deeper understanding of the issues related to switching time will contribute to the refinement of MEMS switch technology, unlocking new possibilities for their integration in emerging electronic systems.

Reducing switching time in MEMS (Micro-Electro-Mechanical System) switches is a multifaceted challenge that researchers address through various innovative methods. One key approach involves optimizing the mechanical design and materials used in MEMS switches. For example, employing advanced materials with superior mechanical properties, such as a low Young’s modulus and high tensile strength, can enhance the response time by minimizing structural deformations during actuation (Zhang et al. 2019 [157]). Furthermore, researchers explore novel actuation mechanisms to achieve faster switching speeds. Leveraging electrostatic, piezoelectric, or magnetic actuation methods allows for rapid and precise control over the MEMS switch, contributing to reduced switching times (Olivieri et al. 2020 [158]). Fabrication techniques also play a crucial role in minimizing switching time. Microfabrication advancements, including improved lithography processes and the use of nanoscale manufacturing technologies, enable the creation of MEMS devices with smaller dimensions and reduced mass, resulting in faster response times (Deng et al. 2021 [125]). Additionally, active control strategies are investigated to dynamically optimize the switching performance of MEMS switches. Closed-loop feedback systems, employing sensors to monitor and adjust the actuation process in real time, contribute to achieving faster and more reliable switching (Wu et al. 2018 [156]). These research endeavors collectively contribute to the ongoing improvement of MEMS switch technology, addressing the challenges associated with switching time and paving the way for their enhanced performance in various applications.

Further, utilizing advanced materials with superior mechanical properties and employing state-of-the-art nanofabrication techniques can contribute to faster switching times [159]. The topology optimization involves systematically designing the structure of MEMS switches to enhance their performance. Liang et al. (2020) provide insights into how topology optimization can be applied to MEMSs for improved functionality, including reduced switching times [160]. Investigating energy-efficient actuation mechanisms is crucial for reducing power consumption and achieving faster switching times. Tang et al. (2022) discusses low-voltage actuation and high-speed MEMS switch design for millimeter-wave applications [161]. Integrating machine learning algorithms and advanced control strategies can enable the real-time adaptive control of MEMS devices, optimizing their response and potentially reducing switching times. Zhang et al. (2021) explore an adaptive control strategy for improving the transient performance of MEMS sensors based on an extreme learning machine [162].

### 4.4. Fabrication Issues in MEMS Switches

Fabrication issues in MEMS (Micro-Electro-Mechanical System) switches can significantly impact their performance, reliability, and commercial viability [163,164,165]. The following are some key fabrication issues commonly encountered in MEMS switches:

#### 4.4.1. Material Selection

**Challenge:** Choosing suitable materials with the right mechanical, thermal, and electrical properties is crucial. The material must also be compatible with the fabrication process. 

**Impact:** Inappropriate material choices can lead to issues such as thermal expansion mismatch, poor electrical conductivity, or susceptibility to stiction.

#### 4.4.2. Stiction and Wear

**Challenge:** Stiction, the undesired adhesion of moving parts, and wear due to friction during actuation are common challenges in MEMS switches.

**Impact:** Stiction and wear can degrade the performance of the switch over time, leading to malfunction or failure.

#### 4.4.3. Process Variability

**Challenge:** Fabrication processes for MEMS devices are sensitive to variations, affecting device performance and yield.

**Impact:** Process variability can lead to inconsistencies in switch characteristics, making it challenging to achieve uniform and predictable behavior across a batch of devices.

#### 4.4.4. Packaging Challenges

**Challenge:** MEMS switches require specialized packaging to protect them from the environment, provide electrical connections, and ensure mechanical stability.

**Impact:** Poor packaging can lead to reliability issues, increased susceptibility to environmental factors, and difficulty in integrating MEMS switches into larger systems.

#### 4.4.5. Energy Consumption

**Challenge:** Achieving low energy consumption during actuation is critical, especially for battery-operated devices.

**Impact:** High-energy consumption can limit the practicality of MEMS switches in energy-sensitive applications and reduce the overall efficiency of the system.

#### 4.4.6. Miniaturization Challenges

**Challenge:** As MEMS devices become smaller, fabrication challenges related to precision and reproducibility become more pronounced.

**Impact:** Inconsistencies in miniaturized structures can lead to performance variations and reliability issues.

#### 4.4.7. Process Integration

**Challenge:** Integrating MEMS switches with other components or technologies in a system can be complex.

**Impact:** Poor integration may hinder the overall functionality of a system or limit the potential applications of MEMS switches.

#### 4.4.8. Reliability and Longevity

**Challenge:** Ensuring long-term reliability and durability is a constant concern in MEMS device fabrication.

**Impact:** MEMS switches should withstand a high number of actuation cycles without degradation to maintain performance over their operational lifespan.

Thus, fabrication plays a vital role in improving performance of the MEMS switches and the aforementioned parameters must be addressed while fabricating an MEMS switch device.

## 5. Summary and Conclusions

A comprehensive review on RF MEMS switches has been presented. The MEMS switches can be classified into various types on the basis of different parameters as discussed in the aforementioned sections. The MEMS switches have been found to be impressive for the desired frequency of operation and applications; still, high-actuation voltage, dielectric charging, stiction, high-switching time are considered major issues in these switches. The methods and procedures introduced by the researchers have helped in improving the performance of the MEMS switchers such as use of low-k suspended metal electrode, methods to reduce the spring constant k of the suspended electrode, size optimization for desired operation at desired frequencies, use of proper dielectric material, and use of different actuation techniques.

The MEMS switches have profound use for high-performing devices such as defense systems, aerospace products, the automobile industry, food industry, and radar communication devices. It has been recommended in different studies by the researchers that the use of graphene can improve the reliability and life span of MEMS switches in future [127,166,167].

## Figures and Tables

**Table 1:** Failure modes and solutions for MEMS switches.

Failure Modes	Solutions
Stiction or adhesion	Implementing anti-stiction coatings, optimizing materials, or employing release mechanisms like electrostatic actuation with pull-in and pull-out voltages.
Contact wear and degradation	Using wear-resistant materials, exploring self-healing materials, and optimizing contact force to minimize mechanical wear.
Dielectric breakdown	Employing high-quality dielectric materials, optimizing switch geometry, and incorporating protective coatings to mitigate breakdown risks.
Residual stress	Designing with balanced stresses, utilizing stress-relief structures, or employing materials with tailored mechanical properties to minimize residual stress.
Electrical overstress (EOS)	Implementing current limiting mechanisms, optimizing materials for electrical robustness, and incorporating protective circuits to prevent EOS-related failures.
Thermal overstress (TOS)	Enhancing thermal management through efficient heat dissipation mechanisms, exploring advanced thermal materials, and optimizing switch design to minimize temperature rise.
Material Fatigue	Investigating materials with high fatigue resistance, optimizing actuation parameters to minimize mechanical stress, and exploring innovative materials like shape memory alloys.

**Table 2:** MEMS switch actuation methods and properties.

Actuation Method	Electrostatic	Electromagnetic	Electrothermal	Piezoelectric	SMA
**Size reduction**	Small	Large	Medium	Medium	Medium
**Contact force**	Generally Low	Moderate	Variable (depends on design)	Moderate	Moderate to high
**Operating voltages**	Low	High	High	Low	High
CMOS integrability	Easy	Difficult	Difficult	Difficult	Difficult to integrate
**Power consumption**	Low to moderate	Variable (depending on design)	Moderate to high	Low to moderate	Moderate to high
**Switching speed**	Fast	Medium	Slow	Fast	Medium

**Table 3:** Performances of different types of RF MEMS switches (2020–2024).

Dimension L × W (μm^2^)	Actuation Mechanism	Contact Type	Actuation Voltage (V)	Power Consumption	Switching Time (ms)	Process Technology	Contact Resistance (Ω)	Reliability in Cycles	Application	Ref. with Year
540 × 330	Electrostatic	ohmic	8 V	—	0.1	Bulk-silicon micromachining process	0.4	5 million	High-performance systems	Li, H. et al. (2020) [58]
—	Thermal actuation	ohmic	—	0.060 w	0.43	Compatible with CMOS technology	100	>5 million	—	Thachil, G. et al. (2020) [76]
400 × 10	Electrothermal and Electrostatic	ohmic	Electrothermal = 7 V electrostatic = 21 V	1 w	70	SOIMUMPS process	1.51–1.81	10 million	High-power applications	Zhu, Y. et al. (2021) [99]
120 × 40	Electrostatic	Capacitive	3	—	—	—	0.15–0.3	—	High-frequency applications	Dalal, K. et al. (2021) [136]
100 × 60	Electrostatic	Capacitive	10.5	—	20	Bulk-silicon micromachining process	—	—	RF applications	Kurmendra et al. (2022) [71]
100 × 21	Electrostatic	ohmic	55	—	—	Silicon-based MEMS switch fabrication	<1	—	THz applications	Feng, Y. et al. (2022) [114]
250 × 150	Electrostatic	Capacitive	9.9	—	33	Micromachining process compatible with conventional IC processes	17	10^9^ switching cycles	—	Wu, Q. et al. (2023) [156]
100 × 160	Electrostatic	ohmic	3.5	—	0.008	Compatible with CMOS technology	—	—	RF signal routing applications	Rajasekhar et al. (2023) [36]
—	Electrostatic	ohmic	50–60	25 dBm	25 μs	Compatible with CMOS technology	—	2.6 billion	—	Jiang, L. et al. (2023) [113]
1000 × 1000	Electrothermal	ohmic	—	—	230 μs	high-g SOI technology	3.5	—	Applications in defense, automobile, and aviation sectors	Vashisth, A. K. et al. (2024) [137]

## Data Availability

Not applicable.
